# Reference dataset for semi-urban and rural Norwegian low voltage distribution grids

**DOI:** 10.1016/j.dib.2025.111453

**Published:** 2025-03-07

**Authors:** Lill Mari Engan, Stine Ekrheim, Sigurd Bjarghov, Jonatan Ralf Axel Klemets, Ivan Schytte, Gerd Kjølle

**Affiliations:** a Department of Energy Systems, SINTEF Energy Research AS, Sem Sælands vei 11, 7034, Trondheim, Norway; b Lede AS, Floodeløkka 1, 3915 Porsgrunn, Norway

**Keywords:** Test grid, Low voltage grid, Grid data, Load data

## Abstract

This paper presents a dataset describing two semi-urban and two rural Norwegian residential low voltage grids. The grids have a radial structure and have a nominal voltage of 230 V. Each grid comes with a corresponding load time series for each consumer with one hour resolution, spanning one year. In order for the dataset to be customizable, the load time series do not contain extra elements such as PV production profiles or electric vehicle charging. On its own, the dataset enables more precise power flow analysis of low voltage distribution grids, but it can also be coupled with medium voltage distribution grids for studies on integrated medium-low voltage distribution systems.

Specifications Table

**Table:** 

Subject	Engineering & Materials science
Specific subject area	Detailed grid and consumer electricity demand data from four residential low voltage distribution grids in the Norwegian power system designed for power flow analyses.
Type of data	Table, Image, Chart, Graph, Figure etc. Raw, Analysed, Filtered, Processed etc. Tables (.csv format).
Data collection	Raw data acquired for a real power distribution system. Anonymized to maintain confidentiality and processed to obtain a simplified reference dataset containing two semi-urban and two rural low voltage grids. The data processing and anonymization procedure is described in detail in the Experimental Design, Materials, and Methods section.
Data source location	Country: Norway. To preserve anonymity, no further details can be specified about the location of the real distribution system from which data were collected, except from in which electricity price area they are located in.
Data accessibility	Repository name: Reference dataset for semi-urban and rural Norwegian low voltage distribution system [1] 10.5281/zenodo.14528192^1^
Related research article	None

## 1 Value of The Data

• The dataset represents four Norwegian residential low voltage distribution grids and were created by the Norwegian research centre CINELDI for use in power distribution research. Low voltage benchmark grids already exist, such as the well-known American and European power system grids from IEEE [2] and CIGRÉ [3]. These, however, are not representative of Norwegian low voltage grids, characterized by long lines. They also do not provide load profiles for a full year, which is useful for analysing the effects of seasonal load variations. As of today, the only publicly available Norwegian low voltage reference grid is from industrial areas [4]. There are no test or reference grids representing residential low voltage distribution systems, like presented in this dataset.
• Norwegian low voltage distribution grids range from compact urban grids to long-distance rural grids, and these will respond differently to changes in loads and production. In general, grids that cover long distances have more significant voltage drops and reduced short-circuit power, while compact grids are more resilient to voltage issues, yet may still be prone to congestion due to thermal limitations. Using one reference grid to represent all low voltage grids would therefore not reflect the variation in conditions in the power grid. Instead, four reference grids have been created: two rural and two semi-urban. Urban grids are less likely to experience issues when transitioning to a decentralized power system and have therefore not been prioritized. Two rural and two semi-urban grids were created to better represent the variation within each category. One of each category represents grids close to voltage problems, while the other two grids are stronger.
• The dataset contains load data with resolution of one hour from real residential areas, representing present-day conditions. The reference grids represent relatively large low voltage grids, but consist of several radials, making it easy to customize the size of the grid as needed. In order to make the reference grids suitable for a variety of research topics, the load profiles do not contain extra elements such as distributed generation or electric vehicle charging.
• The load profiles can be combined with other datasets to research the impact of distributed energy resources, new loads, flexibility resources, and energy storage systems. Distributed generation can be simulated by adding one or more negative loads with the desired production profiles. Tools such as Renewables.ninja [5,6] can be used to generate these production profiles. For residential electrical vehicle charging, datasets of residential charging sessions, such as [7], can be used. By using meteorological data from the same area and time frame, tools like PROFet [8,9] can be used to estimate demands for space heating. It is also possible to use the reference grids for grid planning studies, by simulating the effects of alternatives to grid reinforcement, such as voltage boosters and production curtailment.
• New load and generation can cause unexpected effects in other parts of the distribution system. It can therefore be useful to couple low voltage distribution grids with medium voltage distribution grids. To study these effects, the GitLab repository^2^ includes the example script *merge_mv_lv.py*, which shows how the low voltage grids can be merged with the CINELDI medium voltage reference grid [10] and perform power flow analysis using the Pandapower library [11]^3^. The script includes suggested placement in the medium voltage grid and examples of suitable transformers for each low voltage grid. Since the dataset follows the structure of “MATPOWER case struct”-files (mpc-files) the script can easily be modified to merge with any other medium voltage grid matching the format described in *Dataset*.

## 2 Background

The motivation for compiling this dataset stems from the need to conduct more precise power flow simulations in low voltage distribution grids. In order to conduct realistic analyses on the impact of distributed energy resources in the low voltage grid, proper representation of the low voltage grid is essential. There exist few open-source low voltage grid datasets, and even fewer that contain real electricity demand data in every consumer node from real smart meters for an entire year. In addition, the Nordics, and especially Norway, have a cold climate and high share of electric heating, increasing the importance of a Nordic dataset. Low voltage grids are also typically quite varied, which emphasises the need to separate between rural low voltage grids spanning large distances, from more urban low voltage grids where the distance between consumers is shorter. Since a significant share of new electricity demand, generation and flexibility will be located at the consumer level, detailed low voltage grid datasets can enable higher quality research on the energy transition to a sustainable power system and its impact on distribution grids, such as the quality of supply, reliability, electrical losses and component stress.

## 3 Data Description

In this paper, we present four low voltage reference grids that represent a variety of Norwegian low voltage grids. Two of them are representative of semi-urban grids, while the other two are rural grids. The dataset contains the grid topology and hourly energy demand from each consumer for a full year. The grids are all IT grids with a nominal voltage of 230 V.

### 3.1 Semi-urban grids

This section introduces the semi-urban reference grids. The consumers' building type in the two grids consist mainly of single-family houses, with some townhouses and flats. The distance from the transformer to the farthest consumer is greater than in an urban grid, but shorter than for the rural grids given below. The first semi-urban grid, the 39-bus semi-urban reference grid, is relatively strong, and does not experience large voltage drops, even during peak load. In this context, a strong grid refers to a grid where the voltage drops from the transformer to the most distant buses are limited due to short distances and hence low impedance. Since the thermal capacity of the lines is well below their limits in all cases, only voltage drop is considered when distinguishing between a strong and a weak grid. The second semi-urban grid, the 56-bus semi-urban reference grid, experiences larger voltage drops during peak load, with some consumers approaching the lower legal limit^4^. The load data from the two semi-urban grids are from 2021 and located in price area NO1. The semi-urban grids are constructed by combining two different datasets. The first provides grid topologies with total yearly energy demand, while the second provides load time series data for residential consumers. For each load in the grid, the best load profile is selected based on yearly energy demand, and survey answers, this is explained in more details in *Method: Semi-urban grids*. The reactive power of each load is calculated by assuming a constant power factor of 0.98.

### 3.2 39-bus semi-urban reference grid

**Fig. 1:**
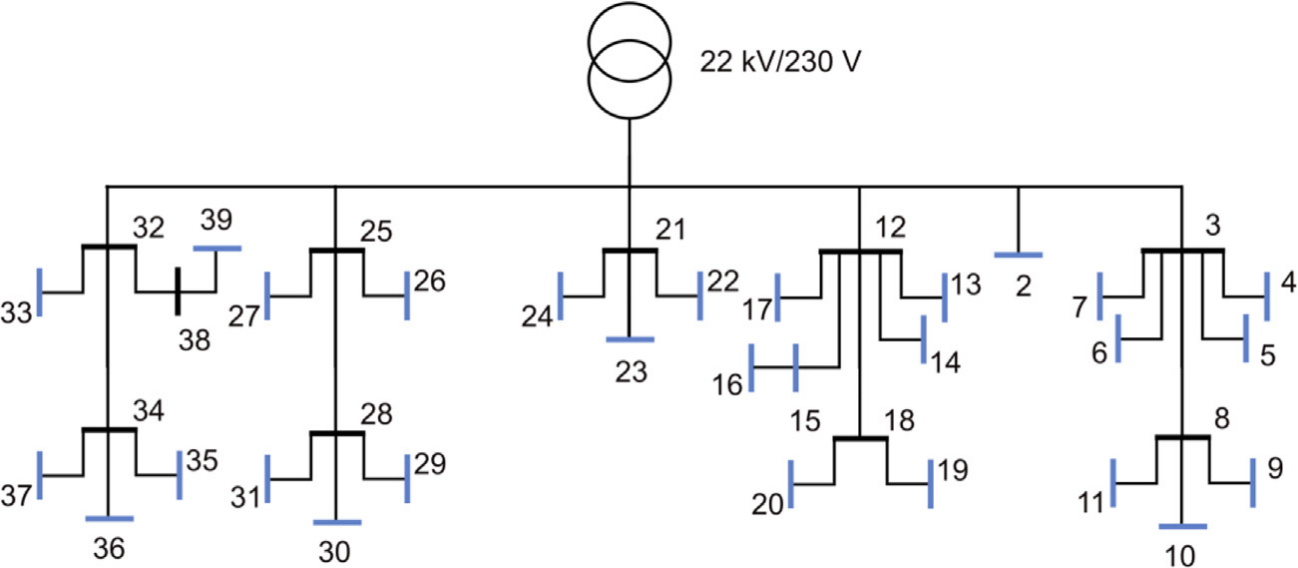
Single line diagram of the 39-bus semi-urban reference grid.

**Fig. 2:**
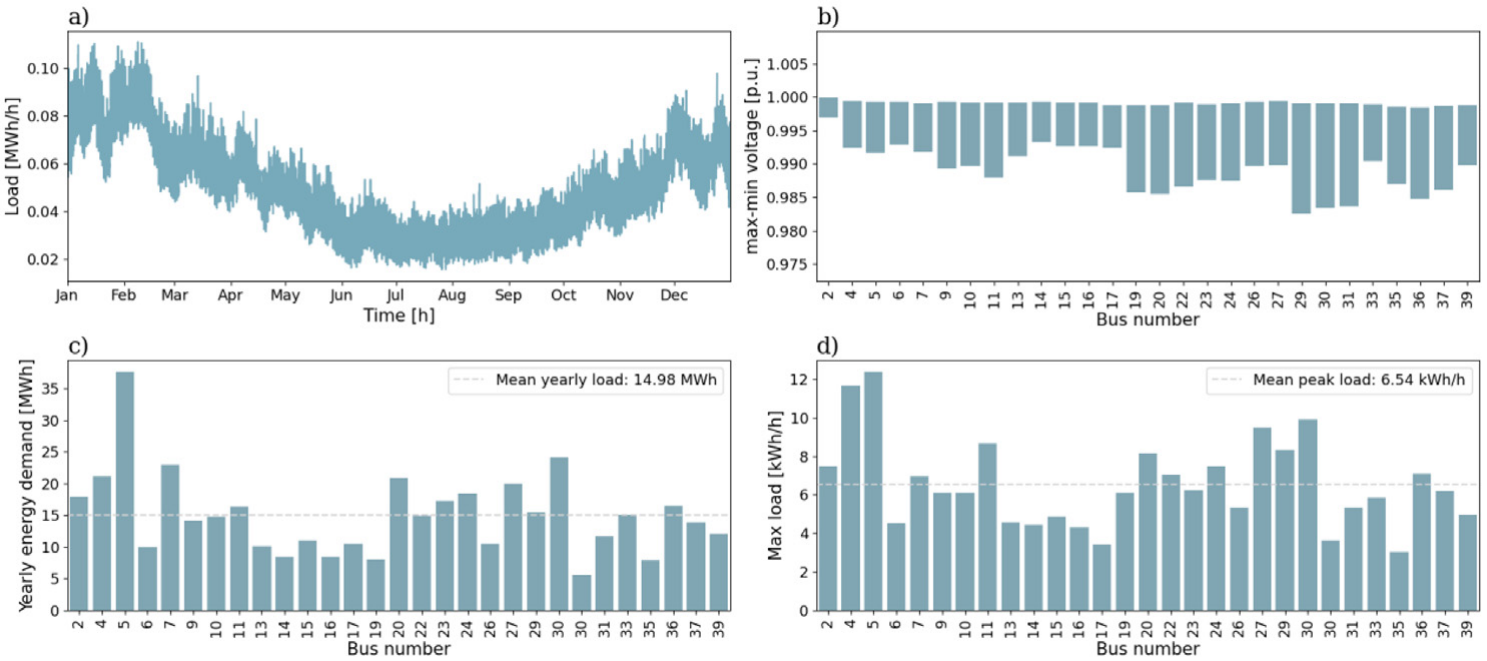
Overview of a selection of grid characteristics for the 39-bus semi-urban reference grid. a) is the load profile, b) shows voltage variation of each load bus throughout the year, while c) shows the yearly energy demand, and d) the peak load, of each load bus.

In Fig. 1 the first semi-urban grid is shown. All blue nodes in the Fig. are load points. It consists of 39 branches and 29 consumers, where 2 of the houses are townhouses and the rest are single-family houses. All the branches in this grid, and the other semi-urban grid, the 56-bus semi-urban reference grid, are underground cables. Fig. 2 shows an overview of the characteristics for the 39-bus semi-urban reference grid. Fig. 2a) gives the aggregated total load for a full year for the low voltage grid. Fig. 2b) gives the voltage variation in per-unit (p.u.) over a full year for all the nodes in the grid. The voltage variation is given as the range from maximum voltage to minimum voltage. The yearly energy demand of all consumers is given in Fig. 2c). The dashed line is the mean yearly energy demand for all the consumers. The peak load for the same group is given in Fig. 2d), and also here the dashed line represents the mean peak value.

The mean annual energy demand for all the consumers is 14.69 kWh and the mean peak load is 6.40 kWh/h. The minimum voltage in the grid is 0.981 p.u.

### 3.3 56-bus semi-urban reference grid

**Fig. 3:**
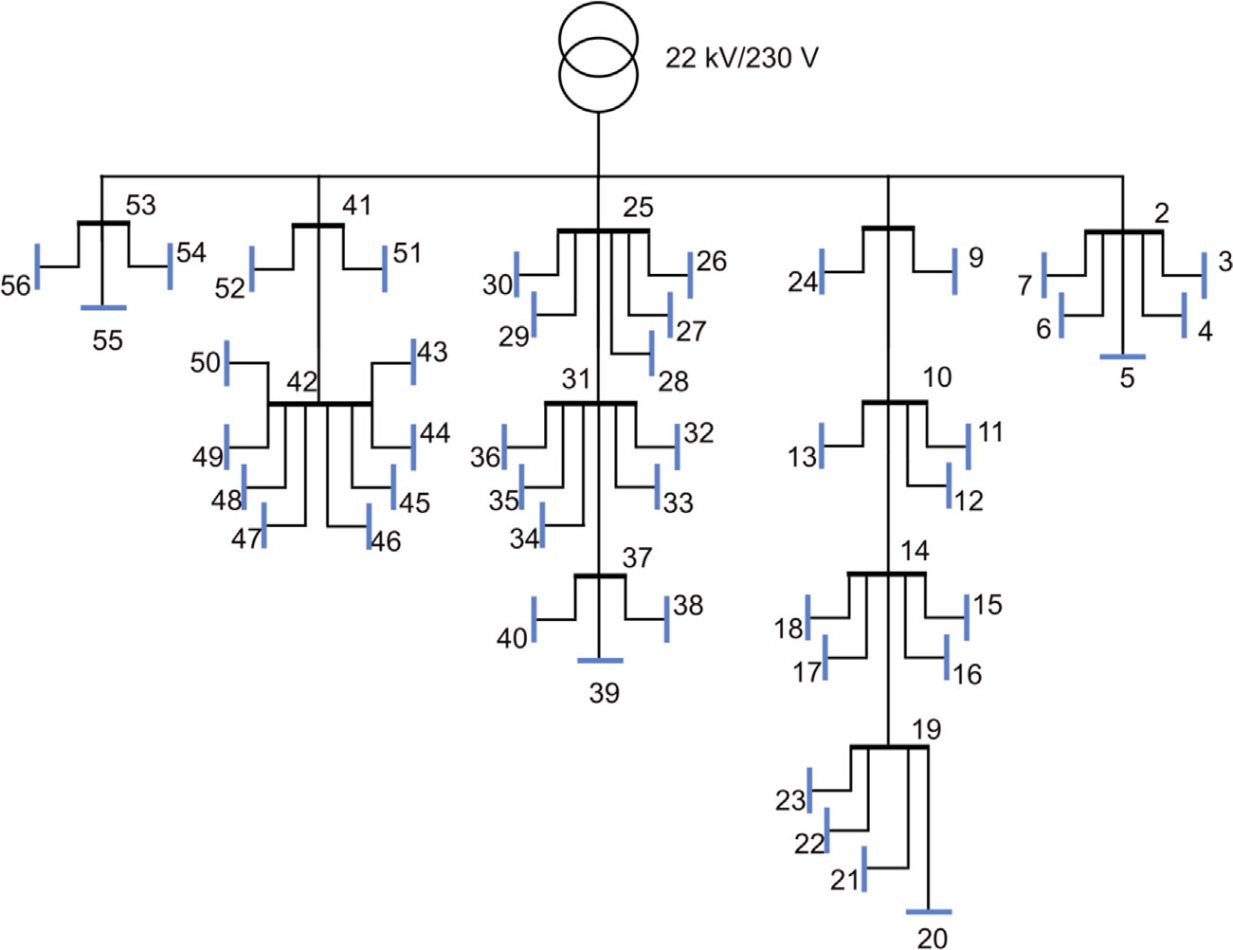
Single line diagram of the 56-bus semi-urban reference grid.

**Fig. 4:**
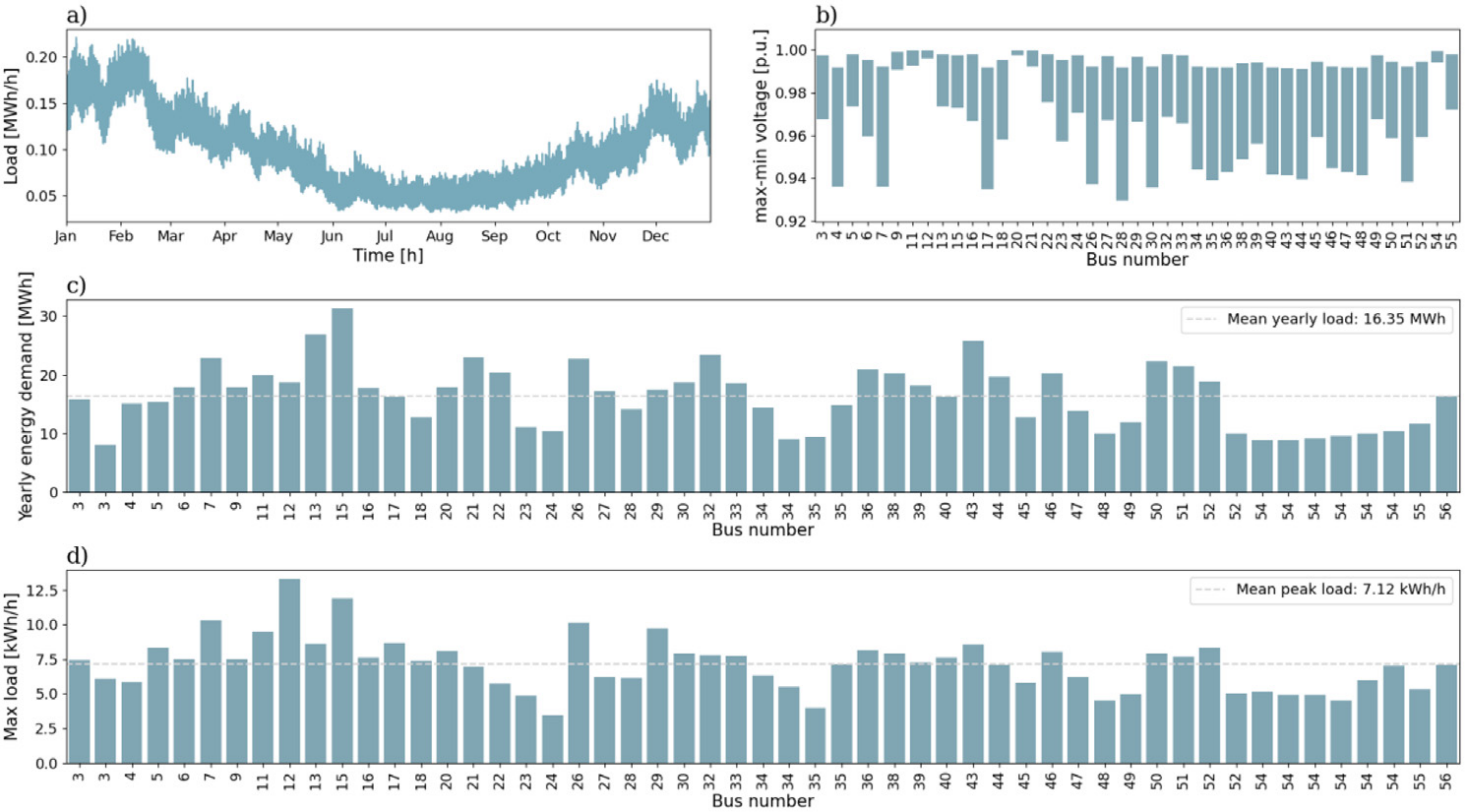
Overview of a selection of grid characteristics for the 56-bus semi-urban reference grid. a) is the load profile, b) shows voltage variation of each load bus throughout the year, while c) shows the yearly energy demand, and d) the peak load, of each load bus.

The second urban grid is shown in Fig. 3 and consists of 55 branches and 53 consumers. For this grid we have 8 townhouses, 6 flats and the remaining are single-family houses. From the figure one can see that there are only 44 load points, the reason for this is that for a load point for a townhouse there are two consumers and for a load point for a flat there are more than two consumers. The aggregated load for the full year is given in Fig. 4a). The yearly energy demand and peak load for all the consumers are given in Fig. 4c) and Fig. 4d), respectively. The mean yearly energy demand in this grid is 16.35 MWh and the mean peak load per consumer is 7.12 kWh/h. The voltage variation for the 56-bus semi-urban reference grid is given in Fig. 4b). The minimum voltage here is 0.927 p.u.

### 3.4 Rural grids

The last two reference grids represent rural Norwegian low voltage grids, characterized by long distances between consumers. The consumer group mostly consists of farms and single-family housing. During peak load, the long power lines result in high voltage drops in the radials. Like for the semi-urban grids, there is one relatively strong grid, the 50-bus rural reference grid, and one weaker grid, the 80-bus rural reference grid. Both grids are weaker than the semi-urban grids from the section above, in the sense that they experience larger voltage drops during peak load. Unlike the semi-urban grids, the rural grids have their original load profiles, but both grid topology and load profiles have been anonymized, as explained in *Method: Rural grids*. It is, however, known that the grid is located in price area NO2, and that the load profiles are from 2021. The remainder of this section describes the rural reference grids in greater detail.

### 3.5 50-bus rural reference grid

**Fig. 5:**
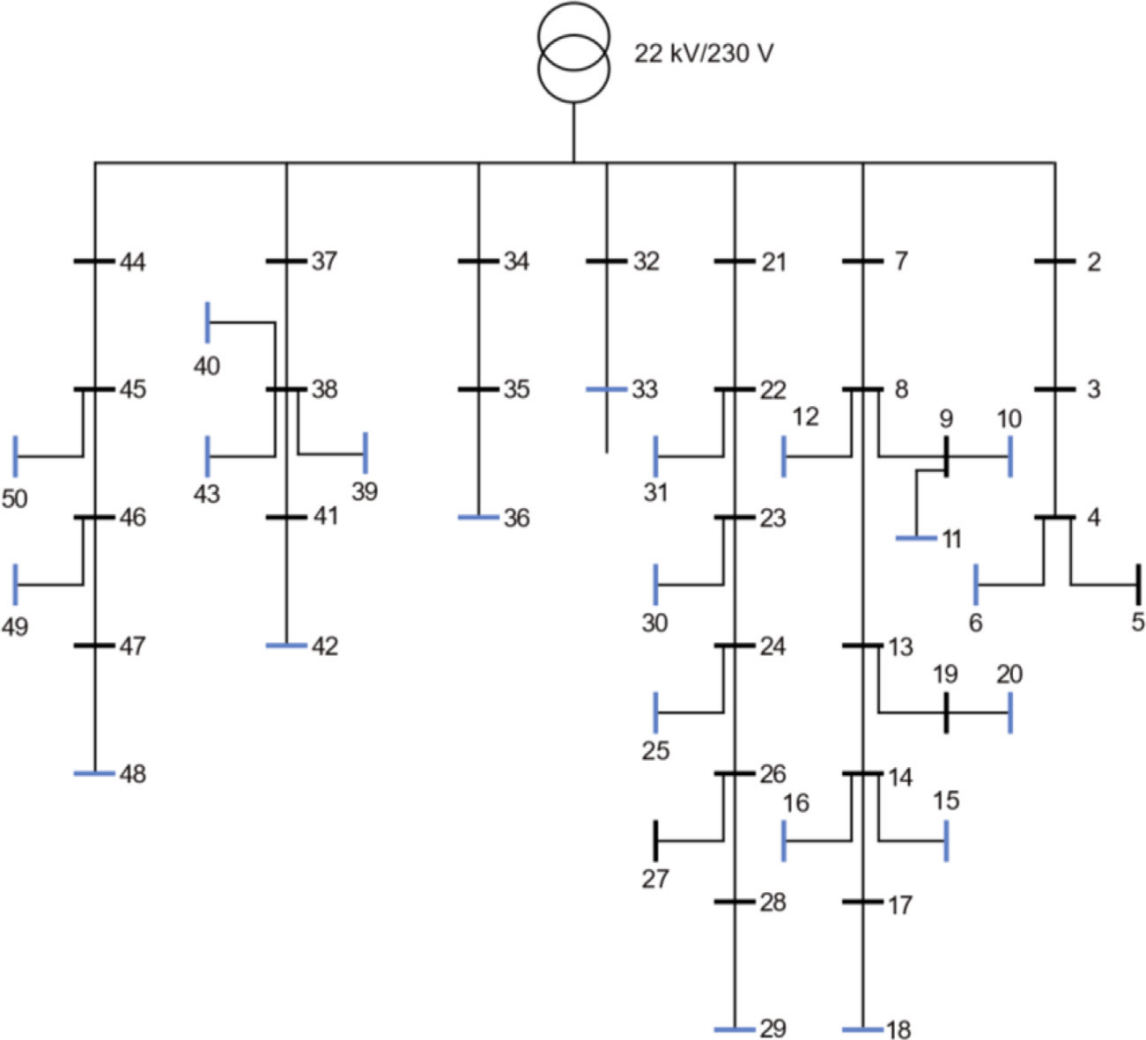
Single line diagram of the 50-bus rural reference grid.

**Fig. 6:**
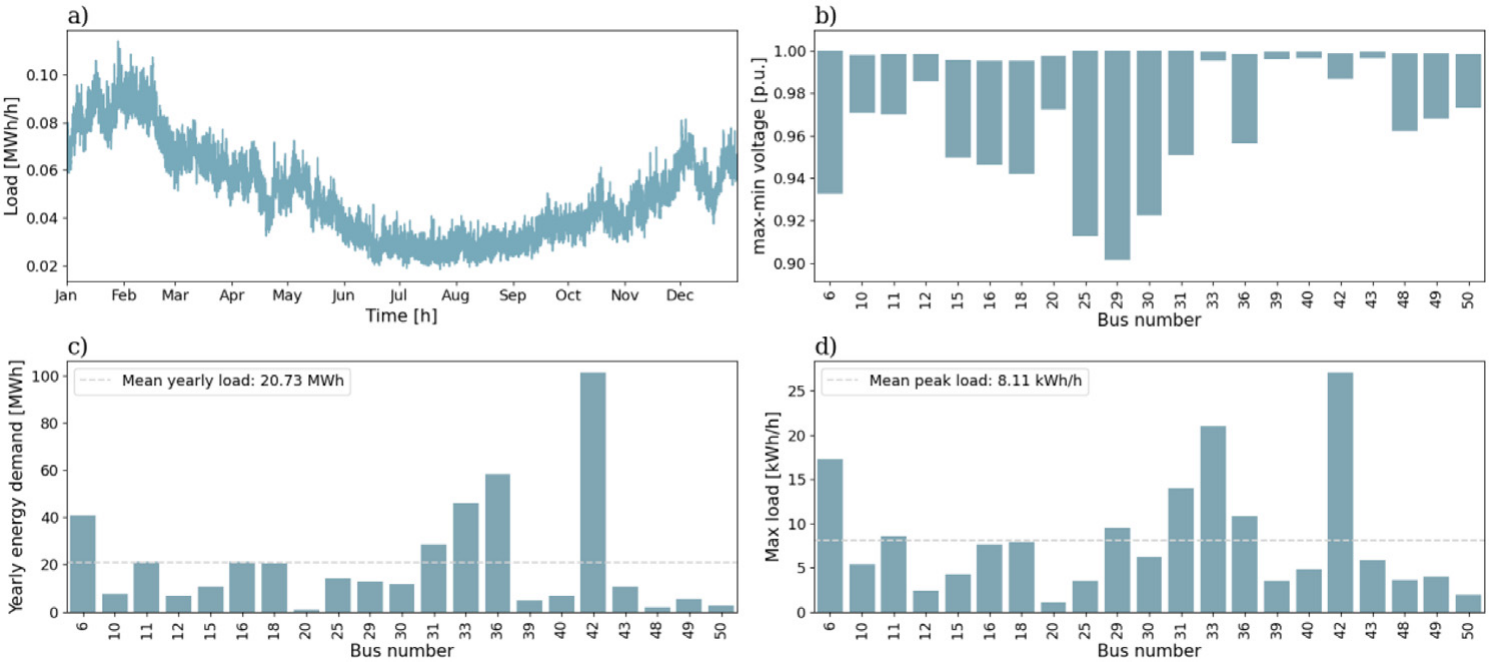
Overview of a selection of grid characteristics for the 50-bus rural reference grid. a) is the load profile, b) shows voltage variation of each load bus throughout the year, while c) shows the yearly energy demand, and d) the peak load, of each load bus.

The 50-bus rural reference grid, shown in Fig. 5 consists of 49 branches and 21 consumers. One of the consumers is an office building, while the remaining consumers represent houses and small farms. Fig. 8 shows the same load characteristics as previously shown for the semi-urban grids. The grid's total yearly energy demand profile, presented in Fig. 6a), follows the seasonal variation expected in Norway, with peaks during winter, and off-peaks during summer. Fig. 6b) shows the yearly voltage variation in each consumer node, and shows that the minimum grid voltage is just above 0.901 p.u. when performing power flow analysis with a feeder voltage of 230 V. FiguresFigure 6c) andFigure 6d) show the yearly energy demand, and peak load, respectively, for each load bus in the grid. The average yearly load is 20.73 MWh, which is higher than the national average for Norwegian households (15.7 MWh in 2023 [13]), but in line with what can be expected in rural areas. The highest peak load is observed at node 42 with a peak of 27 kWh/h, while the average of all peak loads in the grid is 8.11 kWh/h.

### 3.6 80-bus rural reference grid

**Fig. 7:**
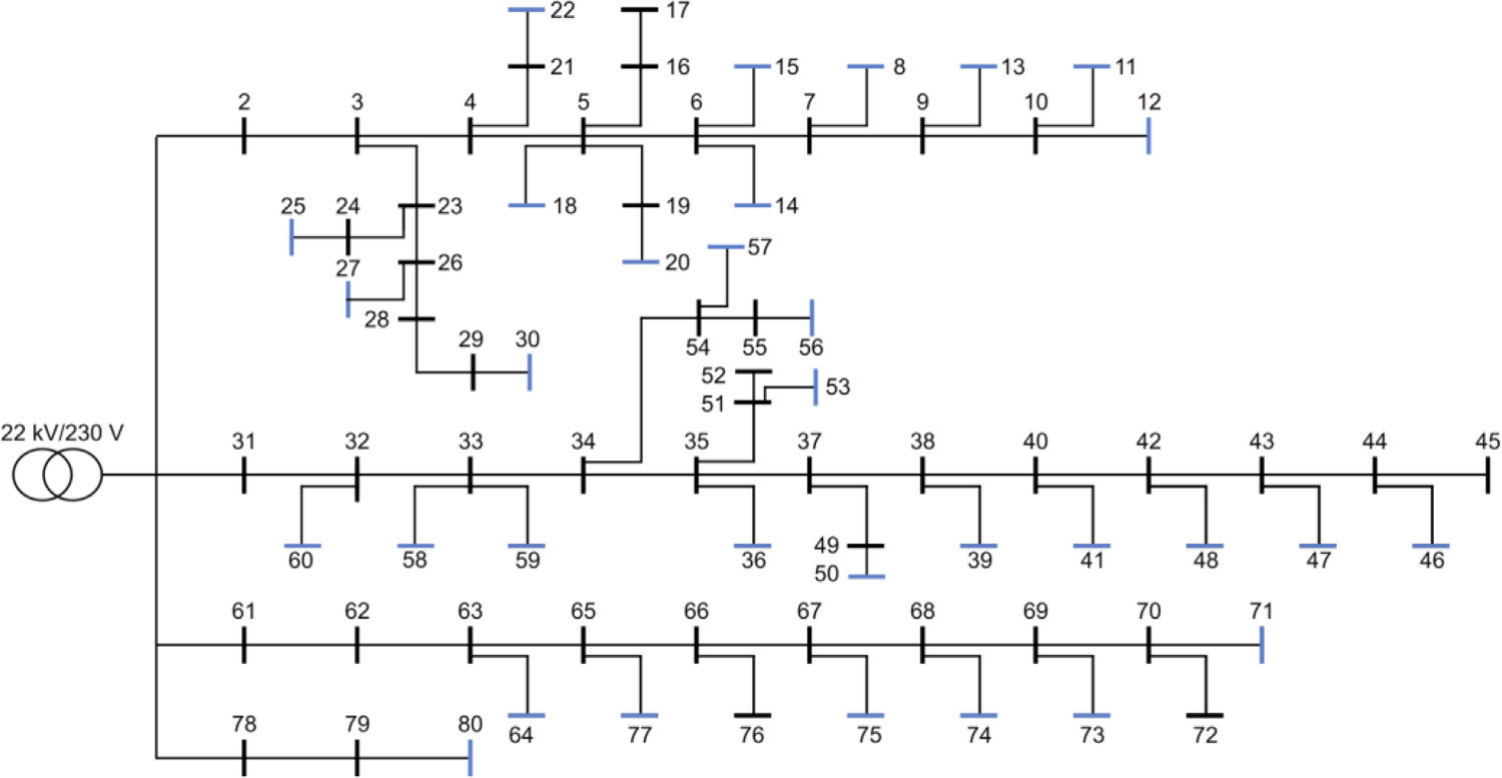
Single line diagram of the 80-bus rural reference grid.

**Fig. 8:**
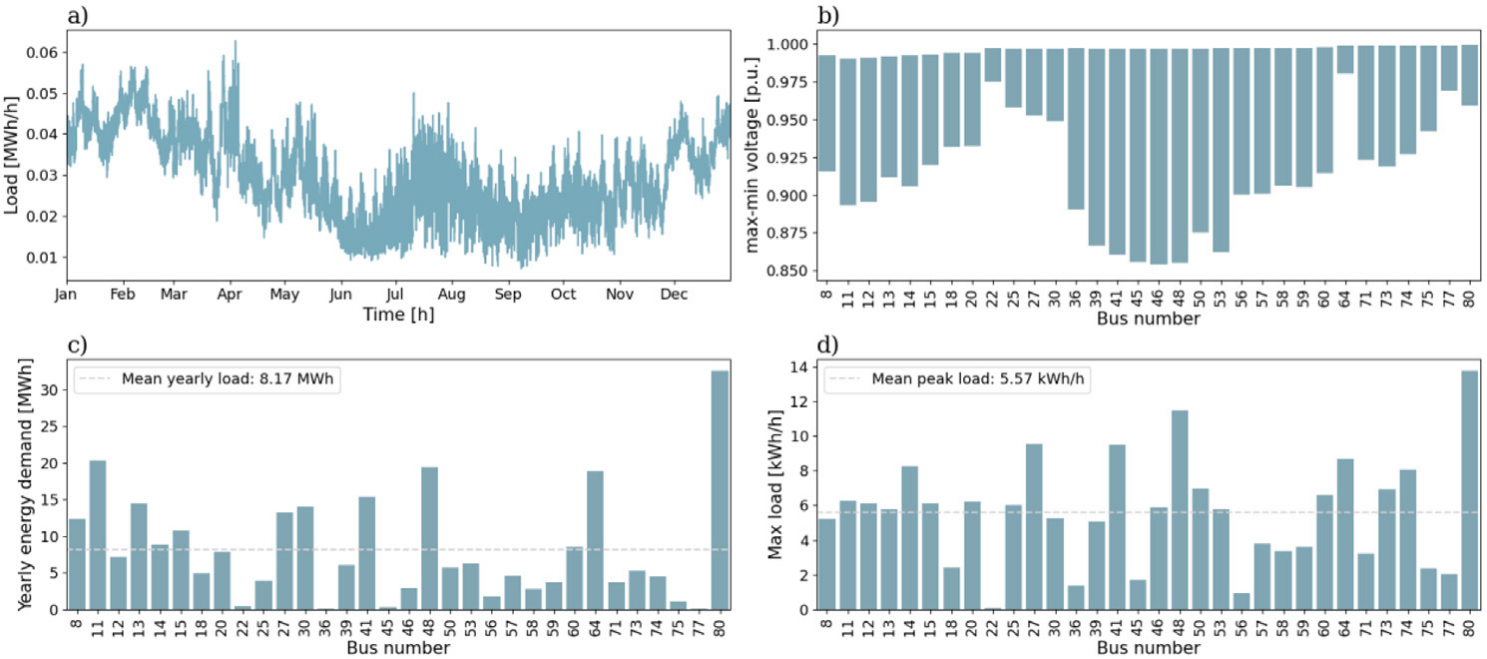
Overview of a selection of grid characteristics for the 80-bus rural reference grid. a) is the load profile, b) shows voltage variation of each load bus throughout the year, while c) shows the yearly energy demand, and d) the peak load, of each load bus.

The 80-bus rural reference grid has a total of 79 branches and 33 consumers. Fig. 7 shows the topology of the 80-bus rural reference grid, which has fewer and longer radials than the 50-bus rural reference grid. Like for the previous grids, Fig. 8 summarizes information about the load profiles.

The 80-bus rural reference grid differs from the other three grids by not exhibiting the same seasonal variation in total energy demand. This is due to the load profiles containing mostly cabins and holiday homes, which results in higher demand during summer and Easter, and lower demand during winter, than what is usually expected. The mean yearly energy demand and mean peak load among the load profiles are 8.17 MWh and 5.57 kWh/h, respectively. When running power flow analysis for the full year, the lowest observed voltage is 0.854 p.u.

### 3.7 Dataset

The dataset contains four folders, one per low voltage reference grid. The name of the folders are *39_bus_semi_urban_grid, 56_bus_semi_urban_grid, 50_bus_rural_grid*, and *80_bus_rural_grid*. For each grid, its topology is defined in the files *mpc_branch.csv, mpc_bus.csv*, and *mpc_base_mva.csv*. Each file corresponds to one of the tables found in mpc-files (“MATPOWER case struct”-files), omitting unnecessary columns [14].

The files *p_load.csv* and *q_load.csv* define load time series for each load in the grid. Finally, additional information about each branch and bus is given in *branch_data_extra.csv* and *load_bus_extra.csv*, respectively. An overview of the files included for each reference grid is given in Table 1. The rest of this section will describe the content of each file in detail.

**Table 1:** Overview of the files describing the different low voltage grids.

File name	Description of data
mpc_branch.csv	Data for the branches in the LV grid on the MATPOWER format
mpc_bus.csv	Data for all the buses in the LV grid on the MATPOWER format
mpc_base_mva.csv	The base power value of the grid
p_load.csv	Hourly time series for active power demand
q_load.csv	Hourly time series for reactive power demand
branch_data_extra.csv	Additional branch information not used for power flow
load_bus_extra.csv	Additional load bus information not used for power flow

### 3.8 Branch data

Branch data can be found in *mpc_branch.csv*. The file describes which buses the branches span between, as well as the impedance and power ratings of the branch. In order to use the files with MATPOWER, additional fields describing angles and power ratings have to be included. Fields that are necessary for MATPOWER, but not for Pandapower, are indicated in the overview given in Table 2. Since the load data has a resolution of one hour, the files only contain a non-zero value for long term power rating (rateA).

**Table 2:** Overview of the data structure in mpc_branch.csv.

Column name	Possible values	Description
fbus	Integer	From bus number
tbus	Integer	To bus number
r	Float	Total branch resistance [p.u.]
x	Float	Total branch reactance [p.u.]
b	Float	Total branch susceptance [p.u.]
rateA	Float	Power rating A (long term rating) [MVA]
rateB†	0	Power rating B (short term rating) [MVA]
rateC†	0	Power rating C (emergency rating) [MVA]
ratio†	0	Transformer phase shift angle [degrees] (positive value means delay)
status	1	Initial branch status (1: in service, 0: out of service)
angmin†	0	Minimum angle difference [degrees]
angmax†	0	Maximum angle difference [degrees]

† For MATPOWER.

### 3.9 Bus data

The file *mpc_bus.csv* includes data about each bus, including bus ID and type. Bus number 1 is always the reference bus (type 3), while all other buses are PQ buses (type 1) with known active and reactive power demands. For buses with associated load time series, the initial active and reactive power demand is included in the bus data. Buses without associated time series always have 0 power demand. Table 3 outlines the columns of the bus data file, where the indicated columns can be omitted for Pandapower, but must be specified for MATPOWER.

**Table 3:** Overview of the data structure in mpc_bus.csv.

Column name	Possible values	Description
bus_i	Integer	Bus number
type	1, 3	Bus type (1: PQ, 2: PV, 3: reference bus, 4: isolated bus)
Pd	Float	Initial active power demand [MW]
Qd	Float	Initial reactive power demand [MW]
Gs†	0	Shunt conductance [MW] (Demanded at V=1.0 p.u.)
Bs†	0	Shunt susceptance [MVar] (MVar injected at V=1.0 p.u.)
area†	1	Same positive integer indicates that the buses are in the same area
Vm†	1	Voltage magnitude [p.u.]
Va†	0	Voltage angle [degrees]
basekV	0.23	Base voltage [kV]
Zone†	1	Loss zone
Vmax	1.1	Maximum voltage magnitude [p.u.]
Vmin	0.9	Minimum voltage magnitude [p.u.]

† For MATPOWER.

### 3.10 Base MVA

For each reference grid, its base power value is given in the file *mpc_base_mva.csv*. The file contains a single value, in the unit MVA. The base power values of each grid is also depicted in Table 4.

**Table 4:** Base power value of all reference grids.

Grid	Base power value
39-bus semi-urban reference grid	0.05 MVA
56-bus semi-urban reference grid	0.05 MVA
50-bus rural reference grid	0.0334 MVA
80-bus rural reference grid	0.0160 MVA

### 3.11 Load data

The load time series for active and reactive load span one year and is given in *p_load.csv* and *q_load.csv*, respectively. Every load node in the grid has a corresponding column in both csv-files, containing hourly load data in the units kWh/h and kVArh/h, respectively. The csv files also have a header row containing the bus number whose load is described by the column. Each row corresponds to the hour of the year, with the first row corresponding to the load data for January 1st, 2021, 00:00 UTC+1, and the last row to December 31st, 2021, 23:00 UTC+1 of the same year. Excluding the header, the csv files have 8670 rows each.

### 3.12 Extra branch data

The dataset also contains files that are not used for power flow analysis but have been included to give additional insight into the distribution grids the dataset represents. Each reference grid includes a file *branch_data_extra.csv*, containing information about branch type and length of each branch. For the semi-urban grids, the branch type is known, while the rural branch types have been estimated based on branch impedance and data from Handbook for grid planning (Planleggingsbok for kraftnett) [15]. Table 5 summarizes the data structure of *branch_data_extra.csv*.

**Table 5:** Overview of the data structure of branch_data_exctra.csv.

Column name	Possible values	Description
fbus	Integer	From bus number
tbus	Integer	To bus number
Length [km]	Float	Length of branch [km]
Branch type	String	Name of branch type

### 3.13 Extra load bus data

Each reference grid folder includes a file called *load_bus_data_extra.csv*, containing additional information about each load. The format of the files is shown in Table 6. For the rural grids, each load has been categorized as private building or office building, with private building referring to residential houses, holiday homes and farms. For the semi-urban grids, the additional ID column indicates which load profile from the original load dataset has been used, as described in *Method: Semi-urban grids*.

**Table 6:** Overview of the data structure of load_bus_data_extra.csv.

Column name	Possible values	Description
bus_i	Integer	Bus number
Consumer type	String	Type of consumer on the load bus
ID†	Integer	ID of load data from survey

† The semi-urban reference grids only.

## 4 Experimental Design, Materials and Methods

In this section, it will be explained how the low voltage grids are obtained from the raw data, and how the modified grids have been validated.

### 4.1 Method: semi-urban grids

For the two semi-urban grids, the grid data has been constructed from two open datasets, one that contains the topology of the grid [16] and another that gives the load time series for the consumers [17], as previously mentioned. The dataset containing the topology of the grid contains a medium voltage grid with several low voltage grids. For the reference grids, only two of them are used. The grids were selected based on the criterion of including one grid with a low voltage drop and another with a higher voltage drop. This dataset also contains the yearly energy demand for every consumer. To get the most realistic time series to each consumer, the yearly energy demand is used. The way this is done is by finding the time series where this value is the closest to the one given in the topology. If the value is more than 5 % smaller or larger, a warning is given, and the time series with annual energy within 10 % is matched. This is the case for four consumers in the 39-bus semi-urban reference grid and two consumers in the 56-bus semi-urban reference grid. There are no consumers that have no matching annual energy within these limits.

In addition to matching the yearly energy demand, housing type is also considered. This is possible because the dataset containing load data is accompanied by a survey, which includes a question on housing type. Load points that have only one consumer are matched with the consumers that live in a single-family house. Load points having two consumers are associated with townhouses, while load points with more than two consumers are matched with consumers with a flat. To maintain the flexibility in the dataset, only consumers without electrical vehicles and PV systems are selected. In addition, all the consumers in the semi-urban grids are located in Oslo, and the year of the time series is 2021.

Since both the topology and load data for the semi-urban grid already are anonymized, no further anonymisation has been conducted.

### 4.2 Method: rural grids

The grid and load data used for the two rural grids were based on raw datasets gathered from the Norwegian distribution grid company Lede. Information about the grids were originally exported as Common Information Models (CIM) whereas the load datasets from 2021 were exported in .xlsx-format. Grid data such as branch impedance, branch length, and bus connections were extracted from the CIM-files to create a model of the grid [18]. Additionally, the ID numbers of the consumers associated with the different nodes were also initially extracted to be able and connect the loads to the correct nodes. However, all ID numbers (for the consumer and substation) were then removed and replaced with a generic bus numbering (1, 2, 3, 4, etc.) to maintain anonymity of the grid and the consumers.

### 4.3 Validation of dataset

After constructing the complete dataset of the low voltage grids, the dataset was evaluated by two industry experts on low voltage grids at Glitre Nett and Lede — two distribution grid companies in Norway. The grids were presented and approved as sound examples of grids with different levels of challenges related to voltage drops and potential congestions. As previously mentioned, some of the grids represent reference grids for low voltage grids close to their limits, and not as “average” Norwegian low voltage distribution grids.

## Limitations

There were some limitations faced when obtaining the low voltage reference grid. The analysis relied on available datasets for low voltage grids, which limited the number of grids to choose from. As a result, the dataset is more representative of the areas the raw data is from, which should be kept in mind when conducting analyses. As an example, will residential areas in northern Norway statistically have a higher load during winter, than the load time series used in the reference grids. To address this issue, two Norwegian DSOs reviewed the grids, however both DSOs operate in the southern region of Norway, which may introduce a regional bias.

## Ethics Statement

The raw load data and grid data have either been obtained from open sources or been anonymized so that it is not possible to identify individual consumer or grid components.

## CRediT Author Statement

**Lill Mari Engan:** Data curation, Formal analysis, Investigation, Methodology, Software, Visualization, Writing – original draft. **Stine Ekrheim:** Data curation, Formal analysis, Investigation, Methodology, Software, Visualization, Writing – original draft. **Sigurd Bjarghov:** Conceptualization, Writing – original draft. **Jonatan Klemets:** Data curation, Writing – original draft. **Ivan Schytte:** Data curation, Validation, Writing – review & editing. **Gerd Kjølle:** Conceptualization, Funding acquisition, Project administration, Supervision, Validation, Writing – review & editing.

## Data Availability

ZenodoReference dataset for semi-urban and rural Norwegian low voltage distribution system (Original data).

## Footnotes

1 GitLab repository: https://gitlab.sintef.no/cineldi_wp6_lv/cineldi_lv_reference_system.

2 See footnote 1.

3 Technical requirements for running the script can be found in the GitLab repository under requirements.txt.

## Footnotes

4 The low voltage limit refers to the Norwegian regulation given in [12], which states that the voltage delivered to a consumer should not drop below 90 % of the nominal level.
